# Characterization of a *Bacillus thuringiensis* chitinase that binds to cellulose and chitin

**DOI:** 10.1186/s13568-017-0352-y

**Published:** 2017-02-28

**Authors:** Shotaro Honda, Toshiyuki Kunii, Kenta Nohara, Satoshi Wakita, Yasusato Sugahara, Masao Kawakita, Fumitaka Oyama, Masayoshi Sakaguchi

**Affiliations:** 1 Department of Chemistry and Life Science, Kogakuin University, 2, 665-1 Nakano-cho, Hachioji, Tokyo 192-0015 Japan; 2 Stem Cell Project, Tokyo Metropolitan Institute of Medical Science, 2-1-6 Kami-kitazawa, Setagaya-ku, Tokyo 156-8506 Japan

**Keywords:** *Bacillus thuringiensis*, Cellulose-binding module, Characterization, Chitinase, Chitin, Expression

## Abstract

*Bacillus thuringiensis* is a Gram-positive soil bacterium that is known to be a bacterial biopesticide that produces insecticidal proteins called crystal proteins (Cry). In the insecticidal process, chitinases are suggested to perforate the peritrophic membrane barrier to facilitate the invasion of the Cry proteins into epithelial membranes. A chitinase gene from *B. thuringiensis* was successfully expressed in a soluble form in *Escherichia coli*, and the gene product was purified and characterized. The purified recombinant enzyme, BthChi74, hydrolyzed an artificial substrate, 4-nitrophenyl *N*,*N*′-diacetyl-β-d-chitobioside [4NP-(GlcNAc)_2_], and the natural substrates, colloidal chitin and crystalline α-chitin, but it did not hydrolyze cellulose. BthChi74 exhibited catalytic activity under a weakly acidic to neutral pH range at 50 °C, and it was stable over a wide pH range for 24 h. Differential scanning fluorimetry (DSF) indicated a protein melting temperature (*T*_m_) of 63.6 °C. Kinetic analysis revealed *k*_cat_ and *K*_M_ values of 1.5 s^−1^ and 159 μM, respectively, with 4NP-(GlcNAc)_2_ as a substrate. BthChi74 produced (GlcNAc)_2_ and GlcNAc from colloidal chitin and α-chitin as substrates, but the activity toward the latter was lower than that toward the former. BthChi74 could bind similarly to chitin beads, crystalline α-chitin, and cellulose through a unique family 2 carbohydrate-binding module (CBM2). The structure–function relationships of BthChi74 are discussed in relation to other chitinases, such as *Listeria* chitinase, which possesses a family 5 carbohydrate-binding module (CBM5).

## Introduction

Chitin is a linear polysaccharide that consists of β-(1 → 4)-linked *N*-acetyl-glucosamine (GlcNAc), and it forms the carbohydrate backbone of crustacean and insect exoskeletons. Chitin is also found in the cell walls of fungi and yeasts, and the microfilarial sheaths of parasitic nematodes. Chitin exists as either α- and β-crystalline chitin or colloidal chitin.

Chitinases (EC 3.2.1.14) are carbohydrate degrading enzymes that hydrolyze the β-(1 → 4)-glycosidic linkages in chitin, and are usually composed of a catalytic domain, one or more carbohydrate-binding modules (CBMs), and other modules, such as a fibronectin type III (FNIII) domain, but some chitinases have only a catalytic domain. Based on amino acid sequence similarity of the catalytic domain, chitinases are classified into two different families, GH18 and GH19, in the CAZy database (http://www.cazy.org/, Lombard et al. 2014). They have different three-dimensional structures and act with different catalytic mechanisms (Iseli et al. 1996; Tews et al. 1997). In a wide variety of living organisms, chitinases degrade chitin, which serves as a nutrient that supplies nitrogen and carbon sources to the organisms. Chitinases and chitin-binding proteins also serve as virulence factors for bacteria pathogen to support the infection of non-chitinous mammalian hosts (Tran et al. 2011; Frederiksen et al. 2013).

*Bacillus thuringiensis* is a Gram-positive soil bacterium that is known to be a bacterial biopesticide that produces insecticidal proteins called crystal proteins (Cry). Cry toxins are pore-forming toxins that induce cell death by forming ionic pores on epithelial cell membranes following insertion into the membranes (Bravo et al. 2004). It was proposed that chitinases perforated the peritrophic membrane barrier, consisting of a network of chitin embedded in a protein-carbohydrate matrix, in the larval midgut and facilitated the invasion of Cry proteins into epithelial membranes (Regev et al. 1996; Ding et al. 2008). However, the expression of chitinase in *B. thuringiensis* is rather low and needs induction by chitin (Thamthiankul et al. 2001; Barboza-Corona et al. 2003; Driss et al. 2005). To gain advantages in the biological control of pests, the constitutive expression of the chitinase at a sufficiently high level and the construction of engineered *B. thuringiensis* strains expressing heterologous chitinase have been reported (Tantimavanich et al. 1997; Thamthiankul et al. 2004; Arora et al. 2003a, b; Barboza-Corona et al. 2003; Driss et al. 2005; Casique-Arroyo et al. 2007; Cai et al. 2007; Okay et al. 2007; Ding et al. 2008).

To further define chitinases that are appropriate to enhance the biopesticidal function of *B. thuringiensis*, comprehensive studies on the enzymatic properties of *B. thuringiensis* chitinase with respect to other related enzymes are needed. Recently, we reported the characteristics of two chitinases, LinChi78 and LinChi35, from *Listeria innocua* (Honda et al. 2016), which are very similar to the *L. monocytogenes* chitinases LmChiB and LmChiA, respectively (Chaudhuri et al. 2010, 2013). In this study, based on knowledge about chitinases of related bacteria, we cloned a chitinase-like gene from *B. thuringiensis* Berliner, expressed it in *Escherichia coli*, and investigated the characteristics of the gene products, namely BthChi74.

## Materials and methods

### Bacterial strains and growth media

*Bacillus thuringiensis* Berliner (serovar israelensis) genomic DNA (ATCC No. 35646 D-5) was purchased from the American Type Culture Collection (ATCC, Manassas, VA, USA). The *E. coli* strains and mediums used as the expression host and the gene engineering host were described previously (Sakaguchi et al. 2015).

### Gene cloning and chemical reagents

Genetic engineering experiments were performed essentially as described by Sambrook and Russell (2012). Colloidal chitin was prepared according to the method of Shimahara and Takiguchi (1988). *N*-acetyl chitooligosaccharides, chitin beads, cellulose, and other reagents were obtained as previously described (Honda et al. 2016).

### Cloning of the chitinase gene from *B. thuringiensis* and expression-vector constructions

To amplify the *B. thuringiensis* chitinase gene, PCR reactions were carried out essentially as previously described (Sakaguchi et al. 2015), using *B. thuringiensis* Berliner genomic DNA as a template and 5′-CGGAATTCCGATGGCTATGAGGTCTCAAAAAT-3′ and 5′-ACGCGTCGACGTTTTCGCTAATGACGGCATTTAAA-3′ (the *Eco*RI and *Sal*I restriction sites are underlined) purchased from Sigma-Aldrich Life Science (Hokkaido, Japan) as the forward and reverse primers, respectively. The reverse primer was designed to fuse a histidine tag [(His)_6_-tag] to the C-terminal end of the recombinant protein when cloned into the pET21d (+) vector (Novagen, Madison, WI, USA) to give pET-BthChi74 expression vector. The nucleotide sequences were confirmed by DNA sequencing (Eurofins Genomics, Tokyo, Japan).

The nucleotide sequence of the *BthChi74* gene is available in the DDBJ/EMBL/GenBank database under Accession Number LC194873.

### Expression and purification of BthChi74

BthChi74 was expressed in *E. coli* BL21 (DE3) and purified essentially as described previously with slight modification (Sakaguchi et al. 2015). Briefly, centrifuged cell extract prepared as previously described was dialyzed against 50 mM Tris–HCl, 1 M (NH_4_)_2_SO_4_ (pH 8.0), and hydrophobic chromatography (HiTrap™ Butyl FF; GE Healthcare, Buckinghamshire, UK) was carried out using a 1 to 0 M linear (NH_4_)_2_SO_4_ gradient at pH8.0. Subsequently, after dialysis against 20 mM Tris–HCl (pH 8.5), anion exchange chromatography (HiTrap™ Q HP, GE Healthcare) was performed using a 0 to 1 M linear NaCl gradient in 20 mM Tris–HCl (pH 8.5). The purity of BthChi74 was confirmed by 10% SDS-PAGE, dialyzed against 20 mM Tris–HCl, 0.5 M NaCl (pH 7.5, TS buffer), and stored at 4 °C.

### Enzyme and protein assays

The enzyme activity was determined at 50 °C using a synthetic chromogenic substrate, 4-nitrophenyl *N,N′*-diacetyl-β-d-chitobioside [4NP-(GlcNAc)_2_], at a concentration of 400 μM, as previously described (Honda et al. 2016). The protein concentration was measured as described previously (Sakaguchi et al. 2015).

### pH and temperature dependence of BthChi74 and kinetic analysis

For the determination of the optimal pH and the optimal temperature, the chitinase activity was measured as previously described (Honda et al. 2016) over a pH range of 3.5–8.0 and over a temperature range of 30–70 °C at pH 6.0, respectively.

To determine the pH stability, enzymes were incubated for 24 h on ice at various pH ranging from 3.5 to 11.0, diluted in TS buffer as described previously (Honda et al. 2016), and then the residual activity was determined at pH 6.0. To determine the heat stability, the differential scanning fluorimetry (DSF) experiment was performed in triplicate, as described previously (Sakaguchi et al. 2015).

To estimate the kinetic parameters, *K*_M_ and *V*_max_ of BthChi74 for 4NP-(GlcNAc)_2_ at 50 °C, the initial rates of hydrolysis in 50 mM 2-morpholinoethanesulfonic acid (MES)-NaOH (pH 6.0) were measured in triplicate over a range of 0.05–0.8 mM. The *K*_M_ and *k*_cat_ values were estimated based on a Michaelis–Menten kinetic model, as described previously (Sakaguchi et al. 2015).

### Analysis of products from colloidal chitin, α-chitin, cellulose, and *N*-acetyl chitooligosaccharides

The products from polysaccharides after hydrolysis with BthChi74 were analyzed using colloidal chitin (2 mg/mL), crystalline α-chitin (2 mg/mL), and cellulose (2 mg/mL) as substrates, according to the method as previously reported (Honda et al. 2016). The reaction mixture containing purified BthChi74 (approximately 5 pmol) and an appropriate substrate in 50 mM MES-NaOH (pH 6.0) were incubated at 37 °C for 1, 24, or 72 h. After stopping the reaction by heating at 95 °C for 10 min, the products of the enzyme reactions were fluorescently labeled and analyzed by high-resolution PAGE according to the methods as described by Jackson (1990) and Kimura et al. (2016). *N*-acetyl chitooligosaccharides, glucose, and cellobiose were used as standards.

(GlcNAc)_5_ or (GlcNAc)_6_ (0.8 mM) was hydrolyzed with approximately 400 pmol of BthChi74 in 50 mM MES-NaOH buffer (pH 6.0) at 25 °C for 1 min, and the products were analyzed by HPLC according to the procedure described by Ishisaki et al. (2012) and Honda et al. (2016).

### Binding analysis to chitin beads, α-chitin, and cellulose

Binding analysis to polysaccharides was performed as previously described (Honda et al. 2016).

## Results

### Cloning of the *B. thuringiensis* chitinase gene and its expression in *E. coli*

**Fig. 1:**
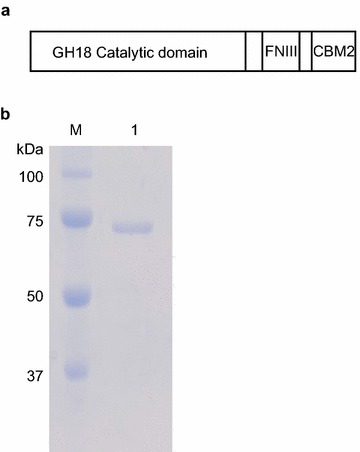
The schematic representation (**a**) and SDS-PAGE analysis (**b**) of BthChi74. **a** BthChi74 is composed of a GH18 catalytic domain, a fibronectin III domain (FNIII), and a family 2 carbohydrate-binding module (CBM2). **b** *Lane M*, molecular weight marker (Precision Plus Protein™ Standards, Bio-Rad laboratories); *lane 1*, purified BthChi74. The numbers in the margin represent the molecular masses (kDa) of the proteins in the molecular weight marker

Figure 1a shows a schematic representation of the recombinant BthChi74 protein. BthChi74 is composed of a GH18 catalytic domain, an FNIII domain, and a CBM2. The BthChi74 protein shows high identity (98%) to BtChiA74 from the *B. thuringiensis* serovar kenyae strain LBIT-82 (Barboza-Corona et al. 2003), with the substitution of 13 amino acid residues within the primary structure. The *BthChi74* gene products were expressed in *E. coli* in an active form, followed by purification to homogeneity through hydrophobic and anion exchange chromatography (Fig. 1b). Purified BthChi74 was detected in SDS-PAGE as a single protein band with an approximate molecular mass of 74 kDa. The expected molecular mass would be 76 kDa, taking the pET vector-derived additional sequences at the N- and C-termini of the gene products into account. The C-terminal side of the gene product might be processed because the gene products could not adsorb to the Ni Sepharose resin through the (His)_6_-tag expected to be attached on the C-terminal side. A similar phenomenon was reported for other chitinases (Watanabe et al. 1994; Thamthiankul et al. 2001).

### The optimal pH and temperature for BthChi74 activity as well as the pH and temperature stability of BthChi74

**Fig. 2:**
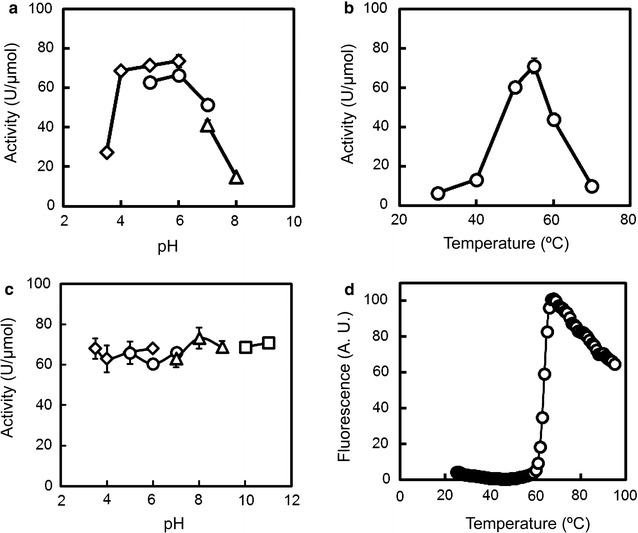
Effects of pH (**a**, **c**) and temperature (**b**, **d**) on BthChi74 activity. **a** The activity was measured at 50 °C in various buffers at different pH values as follows: 50 mM acetate buffer (pH 3.5–6.0, *diamonds*), 50 mM MES-NaOH buffer (pH 5.0–7.0, *circles*), or 50 mM Tris–HCl buffer (pH 7.0–8.0, *triangles*). **b** The activity was measured at various temperatures (30–70 °C). **c** BthChi74 was incubated at 4 °C for 24 h at various pH values as follows: 50 mM acetate buffer (pH 3.5–6.0, *diamonds*), 50 mM MES-NaOH buffer (pH 5.0–7.0, *circles*), 50 mM Tris–HCl buffer (pH 7.0–9.0, *triangles*), or 50 mM carbonate-NaOH buffer (pH 10–11, *squares*). The remaining activity was measured at 50 °C after diluting the pretreated enzymes in TS buffer. The average values with *error bars* are represented as the enzymatic activity. Experiments were performed in triplicate. **d** DSF analysis results for BthChi74 are shown. The experimental procedure is detailed in the “Materials and methods” section. Experiments were performed in triplicate, and the average values are shown

The enzymatic activity as a function of pH and temperature was determined using 4NP-(GlcNAc)_2_ as a substrate, and the results are illustrated in Fig. 2a and b. BthChi74 was optimally active over pH values of 4.0–6.0 at 55 °C. As for the pH and temperature stability, BthChi74 activity was fully retained after treatment for 24 h at 4 °C over the entire pH range tested (pH 3.5–11.5), and DSF analysis gave a *T*_m_ value of 63.6 °C (Fig. 2c, d).

### Kinetic parameters of BthChi74

We determined the steady-state kinetic parameters of BthChi74 with 4NP-(GlcNAc)_2_ as a substrate. The *k*_cat_ and *K*_M_ values of BthChi74 based on the Michaelis–Menten kinetic model were 1.5 s^−1^ and 159 µM, respectively, at 50 °C in 50 mM MES-NaOH buffer (pH 6.0). These values are similar to those of *Serratia marcescens* chitinase B (SmChiB) but slightly different from those of the *Listeria* chitinases LinChi78 and LinChi35 (Table 1).

**Table 1:** Comparison of the kinetic parameters of various chitinases for 4NP-(GlcNAc)_2_

Enzymes	*k*_cat_ (s^−1^)		*K*_M_ (μM)		*k*_cat_/*K*_M_ (s^−1^ μM^−1^)	pH	Temp (°C)
BthChi74	1.5	±0.02	159	±0.5	0.01	6.0	50
LinChi78	10.4	±0.4	127	±0.8	0.08	6.0	50
LinChi35	51.1	±0.02	885	±25	0.06	5.0	50
SmChiB	1.4	±0.5	181	±35	0.01	6.1	37

The kinetic parameters of various chitinases and the conditions for activity measurements were obtained from the following resources: BthChi74 (this study), LinChi78 (Honda et al. 2016), LinChi35 (Honda et al. 2016), and SmChiB (Krokeide et al. 2007)

### Hydrolytic activities of BthChi74 toward colloidal chitin, α-chitin, cellulose, and chitin oligosaccharides

**Fig. 3:**
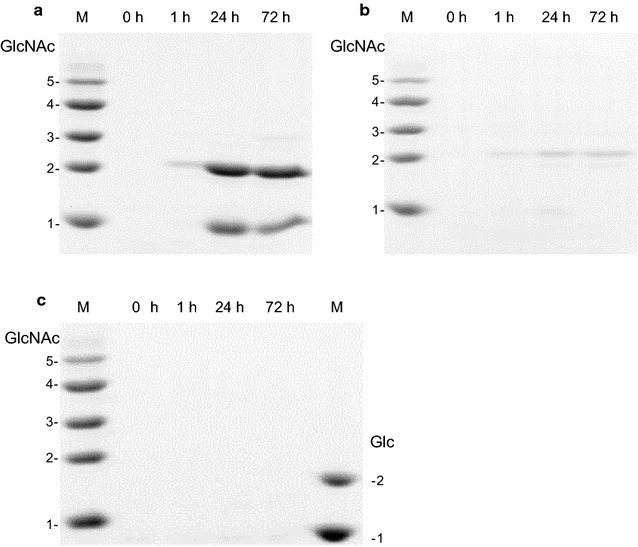
Degradation products from colloidal chitin (**a**), α-chitin (**b**), and cellulose (**c**) after digestion with BthChi74. Reactions were conducted for 1, 24, or 72 at 37 °C. The produced chitin fragments were labeled as described in the “Materials and methods” section and separated by PAGE. *N*-acetyl chitooligosaccharides were used as standards in (**a**), (**b**), and (**c**). Glucose and cellobiose were used as standards in (**c**)

The hydrolytic products from polymeric substrates after BthChi74 digestion were analyzed using PAGE. Figure 3 shows the products from colloidal chitin, α-chitin, and cellulose as the substrates. BthChi74 produced dimer (GlcNAc)_2_ and monomer (GlcNAc), from colloidal chitin (Fig. 3a). Only a very small amount of (GlcNAc)_2_ was produced from α-chitin (Fig. 3b), indicating that it was a poor substrate. BthChi74 was inert toward cellulose under the tested conditions (Fig. 3c).

**Fig. 4:**
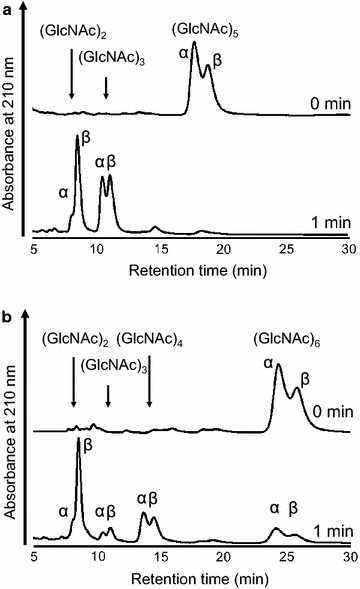
Anomeric analysis of hydrolytic products from (GlcNAc)_5_ (**a**) and (GlcNAc)_6_ (**b**). *Arrows* indicate the eluted positions of GlcNAc_n_ (n = 2–4), and peaks are assigned to the α- and β-anomers

Figure 4a and b shows the hydrolytic products from (GlcNAc)_5_ and (GlcNAc)_6_, respectively, whose initial anomeric ratios (α:β) were approximately 6:4. After the hydrolysis of (GlcNAc)_5_ for 1 min, (GlcNAc)_2_ and (GlcNAc)_3_ were observed and their anomeric ratios were 1:9 and 4.5:5.5, respectively. The products of (GlcNAc)_6_-hydrolysis with anomeric ratios in parentheses were (GlcNAc)_2_ (1.5:8.5), (GlcNAc)_3_ (3:7), and (GlcNAc)_4_ (5.5:4.5) (Fig. 4b). These results suggested that BthChi74 hydrolyzed chitin oligosaccharides at the second and third glycosidic linkage from the non-reducing end with retention of the anomeric configuration. In addition, a high (GlcNAc)_2_/(GlcNAc)_4_ ratio in (GlcNAc)_6_-hydrolysis suggested that BthChi74 adopt processive manner in the substrate degradation, similar to LinChi78 as previously described (Honda et al. 2016).

### Binding of chitinases to chitin beads, α-chitin, and cellulose

**Fig. 5:**
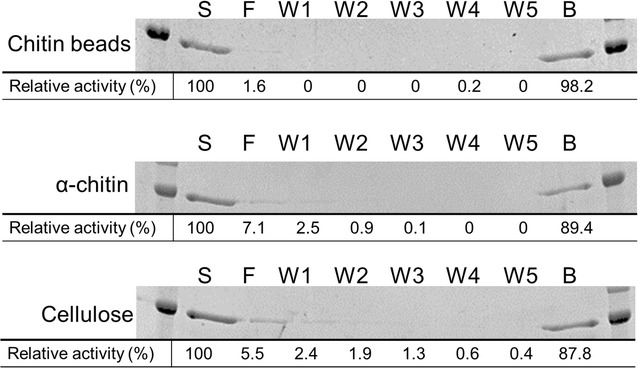
Binding activity of BthChi74 to chitin beads, α-chitin, and cellulose. S, F, W and B denote the starting sample, flow-through fraction, wash fraction and binding fraction, respectively. The values represent the relative activity recovered in each fraction with the initial activity in the starting sample taken as 100%. Experiments were carried out in duplicate, and the average values are indicated

To examine how BthChi74 interacts with various polysaccharides, we performed the binding assay between chitinase and chitin beads, α-chitin, and cellulose (Fig. 5). BthChi74 efficiently bound to chitin beads, α-chitin, and cellulose with binding percentages of 98, 89, and 88%, respectively. The uniform affinity for these polysaccharides is different from the binding properties of LinChi78 (Honda et al. 2016), which showed different affinities of 89, 57, and 31% toward chitin beads, α-chitin, and cellulose, respectively.

## Discussion

The *BthChi74* gene of *B. thuringiensis* was successfully expressed in a soluble and active form in *E. coli*. The product, BthChi74, was purified to homogeneity and then identified as a chitinase of *B. thuringiensis* with a CBM2 module (Fig. 1a). In the CAZy database, CBM2 is mostly added to carbohydrate hydrolases acting on β-(1 → 4)-glycan, i.e., β-(1 → 4)-glucanases, xylanases, or cellulases, although bacterial and archaeal chitinases possessing this module have also been reported (Fujii and Miyashita 1993; Reguera and Leschine 2003; Nakamura et al. 2008).

BthChi74 is a chitinase that hydrolyzes 4NP-(GlcNAc)_2_, colloidal chitin, and α-chitin, but not cellulose. The chitinase was active under weakly acidic to neutral conditions and had a high optimal temperature of 50 °C. The enzyme was stable over a wide pH range for 24 h, and the protein structure was heat-stable with a *T*_m_ value of 63.6 °C. In addition, BthChi74 could bind to chitin beads, α-chitin, and cellulose to similar extents. The reactivity toward chitin polymers together with the stability over a wide pH range is consistent with the potential of BthChi74 to degrade chitinous materials after exposure to the alkaline conditions of the larval midgut.

Barboza-Corona et al. (2003) reported the cloning and heterologous expression in *E. coli* of a chitinase BtChiA74 from the *B. thuringiensis* serovar kenyae strain LBIT-82, with which BthChi74 is 98% identical. They also reported preliminary characterization of the catalytic properties of BtChiA74 in a crude *E. coli* cell extract using an artificial substrate. In the present study, we performed a detailed study of catalytic properties of BthChi74 using an enzyme preparation purified to homogeneity and artificial as well as natural substrates such as colloidal and α-crystalline chitin and cellulose. These results represent refinement and extension of those obtained previously with crude BtChiA74 and may be compared with those of other more distantly related chitinases to help in extending our understanding on chitinases and related enzymes.

**Fig. 6:**
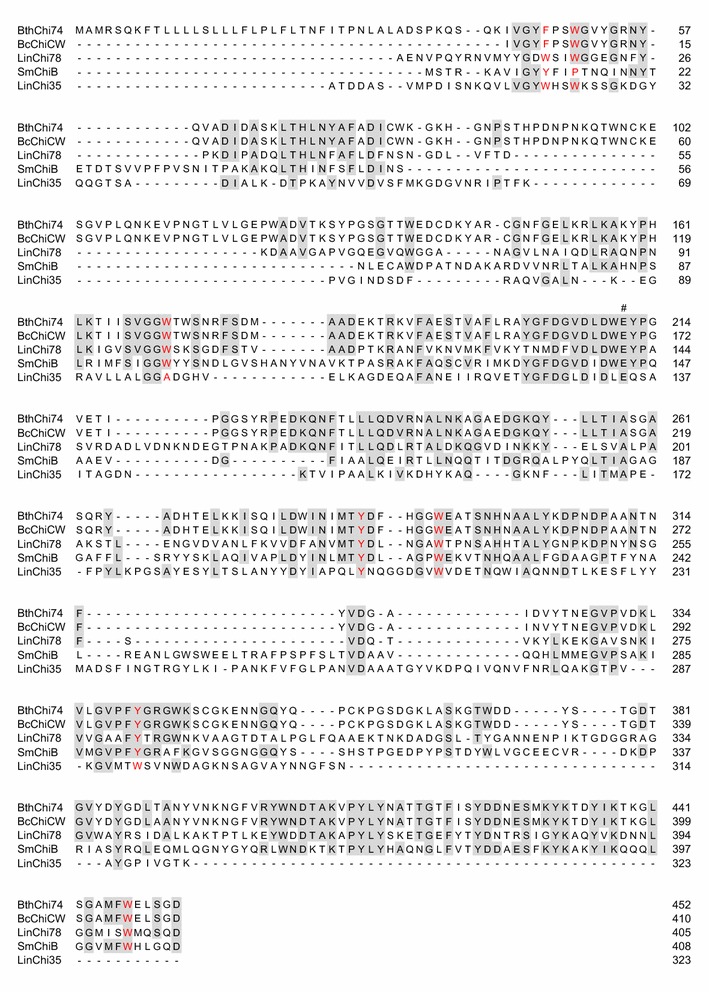
Sequence alignment of the catalytic domain in BthChi74 with those in other GH18 bacterial chitinases. The aligned sequences (Accession Number) are the following: BthChi74 (LC194873); BcChiCW (AAM48520); LinChi78 (LC092875); SmChiB (CAA85292); and LinChi35 (LC092876). The *hash* symbol (#) denotes the putative catalytic residue. The putative amino acid residues interacting with a substrate in subsites as described in *Bc*ChiA (Watanabe et al. 2003) are colored in *red*. Amino acid residues that are identical in three or more of the aligned enzymes are *shaded*

BthChi74 shows 33% identity to LinChi78 within the catalytic domains. The aromatic residues such as Trp and Tyr residues involved in substrate recognition in subsites −5 to +2 of LinChi78 are conserved in BthChi74 (Fig. 6). Among them, Trp residues that play an important role in the processive hydrolysis of the chitin chain and provide the ability to efficiently degrade insoluble chitin are conserved at positions 171 and 292 (Watanabe et al. 2003; Horn et al. 2006). Similar to LinChi78, BthChi74 possesses an α+β insertion in the catalytic domain, which is suggested to be involved in processive and exolytic hydrolysis (Li and Greene 2010). Based on the occurrence of these conserved residues and our results with regard to hydrolysis of chitin oligosaccharides, it is tempting to assume that BthChi74 may be an exo-type chitinase acting in a processive manner, similarly to LinChi78, as previously reported (Honda et al. 2016). However, in the absence of substantial evidence and in view of the observation that the amount of monomer product from colloidal chitin in BthChi74 hydrolysis is slightly higher than that in LinChi78, the validity of this assumption remains to be seen.

CBM2 represents one of the largest CBM families in CAZy and is comprised of members that can bind to cellulose, xylan, and chitin, but it is not common among chitinases. More than half of the approximately 350 chitinases with CBMs possess CBM18 or CBM5, which are the first and second most frequent modules, respectively, and 30 chitinases, which is approximately 10% of CBM-possessing chitinases, have been registered to have CBM2. To date, comparative studies between chitinases with different CBMs are limited.

BthChi74 possesses CBM2 and could bind to chitin beads and the crystalline polysaccharides α-chitin and cellulose, whereas LinChi78 has CBM5 and effectively binds to chitin beads but binds poorly to α-chitin and cellulose compared to BthChi74 (Honda et al. 2016). The differences in their binding abilities may be explained by the intrinsic properties of their respective CBM. The catalytic properties of these enzymes toward the artificial substrate 4NP-(GlcNAc)_2_ were not affected much by different CBMs.

**Fig. 7:**
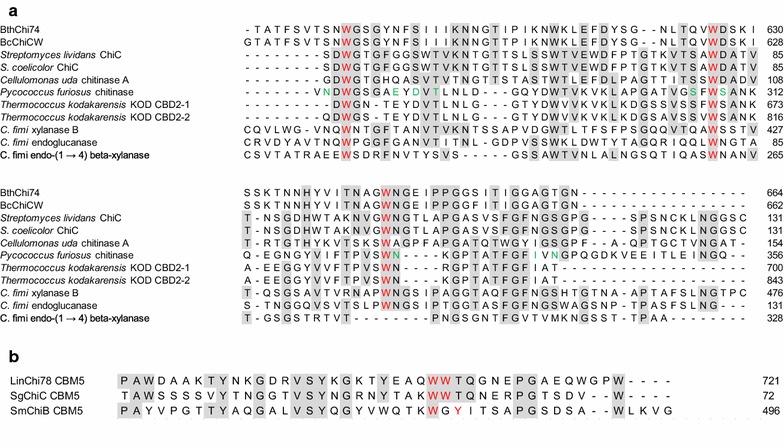
Sequence alignment of CBM2 in BthChi74 (**a**) and of CBM5 in LinChi78 (**b**) with those in other carbohydrate enzymes. **a** The aligned sequences (Accession Number) are the following: BthChi74 (LC194873); BcChiCW (AAM48520); *Streptomyces lividans* ChiC (BAA02168); *S. coelicolor* ChiC (CAB94547); *Cellulomonas uda* chitinase A (AAG27061); *Pyrococcus furiosus* chitinase (AAL81357); *Thermococcus kodakarensis* KOD CBD2-1 (BAD85954); *T. kodakarensis* KOD CBD2-2 (BAD85954); *C. fimi* xylanase B (AEA30147); *C. fimi* endoglucanase (AEE47298); and *C. fimi* endo-(1 → 4) beta-xylanase (CAA54145). The aromatic and other residues putatively interacting with carbohydrates are colored in *red* and *green*, respectively. Amino acid residues that are identical in five or more of the aligned enzymes are shaded. **b** The aligned sequences (Accession Number) are the following: LinChi78 (LC092875); *Streptomyces griseus* ChiC (BAA23739); and SmChiB (CAA85292). The aromatic residues putatively interacting with carbohydrates are colored in *red*. Amino acid residues that are identical in two or more of the aligned enzymes are shaded

**Fig. 8:**
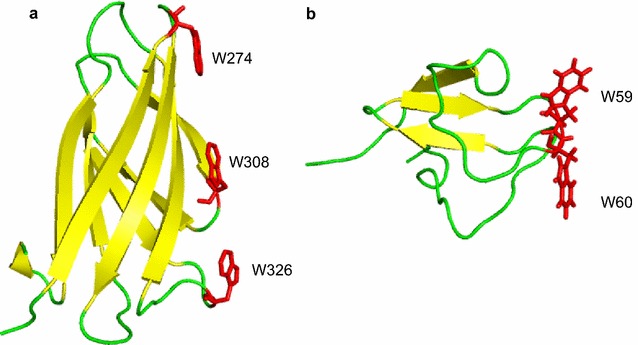
Crystal structures of CBM2 in *Pyrococcus furiosus* chitinase (PDB No. 2CWR, (**a**)) and CBM5 in *Streptomyces griseus* ChiC (PDB No. 2D49, (**b**)). The stick models of the Trp residues involved in binding carbohydrates are colored in *red*

The CBM2 of BthChi74 shows 97% identity to that of the chitinase CW from *B. cereus* (BcChiCW, Huang et al. 2005), 35% to that of the chitinase C from *Streptomyces lividans* (Fujii and Miyashita 1993), 35% to that of the chitinase A from *Cellulomonas uda* (Reguera and Leschine 2003), 32% to that of the endoglucanase A from *C. fimi* (Wong et al. 1986), and 31% to that of the xylanase D from *C. fimi* (Millward-Sadler et al. 1994). CBM2 domains are β-sandwich fold domains, typically comprised of two four-stranded sheets (Xu et al. 1995; Nakamura et al. 2008; Hanazono et al. 2016). In CBMs, it is known that the aromatic rings of surface-exposed Trp residues play important roles in binding carbohydrates by stacking a sugar, mainly through hydrophobic interactions, on a planar and wide surface of the structure (Figs. 7a, 8a; Xu et al. 1995; Nakamura et al. 2008; Hanazono et al. 2016). Such Trp residues, Trp591, Trp626, and Trp645, are conserved and could be exposed on the surface of the CBM2 of BthChi74. In contrast, the CBM of LinChi78 belongs to family 5 (CBM5) and shows 46% identity to *Streptomyces griseus* chitinase C (SgChiC), whose catalytic domain is classified into the GH19 family. In CBM5 of SgChiC, two Trp residues are conserved and function in carbohydrate binding on the surface of the domain composed of a triple antiparallel β-sheet (Akagi et al. 2006; Itoh et al. 2006; Kezuka et al. 2006). The alignment analysis indicated that CBM5 of LinChi78 has corresponding residues at the 706th and 707th positions, and these residues could be involved in binding chitin on a narrow area, similarly to those of SgChiC, as previously reported (Figs. 7b, 8b; Akagi et al. 2006; Itoh et al. 2006).

Nakamura et al. (2008) substituted two acidic residues, Glu and Asp, located near the first Trp residue in the CBM2 of *Pyrococcus furiosus* chitinase for Thr and Asn, respectively, which are observed in the corresponding positions in CBM2 of the endoglucanase from *C. fimi.* The double mutant acquired a significant binding affinity to cellulose, which was lacking in wild-type *P. furiosus* chitinase, without the loss of affinity to chitin. It is interesting that Asn and Ser are found in the CBM2 of BthChi74 at the positions corresponding to Thr and Asn of *C. fimi* endoglucanase (Fig. 7a). Our present results strengthen the notion that acidic groups at these Trp neighbors somehow restrict efficient cellulose binding, although additional studies are needed in the future to confirm the functions of these residues.

The reason why *B. thuringiensis* chitinase which could not hydrolyze cellulose possesses CBM2 and could bind to cellulose remains unknown. Similarly puzzling is how chitinases of *Listeria monocytogenes* are intimately involved in the pathogenesis of human listeriosis. How chitinases can promote pathogenicity in animal hosts without a chitinous surface remains unclear, but the involvement of bacterial chitinases and carbohydrate binding proteins in virulence have been reported (Tran et al. 2011; Frederiksen et al. 2013). It may be relevant that the activity of *Listeria* chitinase on non-chitinous substrates has been reported. Similarly, it is possible that *B. thuringiensis* chitinase may act on a crystalline chitinous matrix and on an unidentified substrate, which is related to its cellulose binding capacity. This is one of the obvious challenging tasks that awaits investigation. However, the basic enzymatic properties of chitinases elucidated in the present study will extend our understanding of the relationship between the structure and function of chitinases, and they will not only help in defining the exact functional roles of chitinases in the invasion of other organisms but also in making chitinases useful in biotechnological applications.

## Abbreviations

ATCC: The American Type Culture Collection
CBM: carbohydrate-binding module
Cry: crystal protein
DSF: differential scanning fluorimetry
FNIII: fibronectin type III
GlcNAc: *N*-acetyl-glucosamine
(His)_6_-tag: histidine tag
MES: 2-morpholinoethanesulfonic acid
PAGE: polyacrylamide gel electrophoresis
SDS-PAGE: sodium dodecyl sulfate–polyacrylamide gel electrophoresis
*T*_m_: protein melting temperature
4NP-(GlcNAc)_2_: 4-nitrophenyl *N*,*N*′-diacetyl-β-d-chitobioside
